# RETRACTION: Protective Effects of Celastrol on Diabetic Liver Injury Via TLR4/MyD88/NF‐*κ*B Signaling Pathway in Type 2 Diabetic Rats.

**DOI:** 10.1155/jdr/9840673

**Published:** 2026-03-23

**Authors:** Journal of Diabetes Research

RETRACTION: L‐P. Han, C‐J. Li, B. Sun, Y. Xie, Y. Guan, Z‐J. Ma, L‐M. Chen, “Protective Effects of Celastrol on Diabetic Liver Injury via TLR4/MyD88/NF‐*κ*B Signaling Pathway in Type 2 Diabetic Rats”, *Journal of Diabetes Research*, 2016, 2641248, https://onlinelibrary.wiley.com/doi/10.1155/2016/2641248

The above article, published online on 19 January 2016 in Wiley Online Library (wileyonlinelibrary.com), has been retracted by agreement between the journal Associate Editor, Bright Starling Emerald, and John Wiley & Sons Ltd. The retraction has been agreed due to errors identified in the figures. Specifically:

• In Figure 2b, the DM + CM panel is incorrect.
• The p‐IkBa blots shown in Figure 3c appear identical to the α‐SMA bands shown in Figure 2b of [1]
• The B‐Actin blots shown in Figure 3c appear identical to the B‐Actin bands shown in Figure 2b of [1]
• The B‐actin blots shown in Figures 4c and 4d appear similar

After assessment of the concerns and author’s original images, the editorial board has lost confidence in the results and conclusions presented, and the article is retracted.

The authors agree to the retraction.

## References

[1] Ma Z. J. , Zhang X. N. , Li L. , Yang W. , Wang S. S. , Guo X. , Sun P. , and Chen L. M. , Tripterygium Glycosides Tablet Ameliorates Renal Tubulointerstitial Fibrosis via the Toll-Like Receptor 4/Nuclear Factor Kappa B Signaling Pathway in High-Fat Diet Fed and Streptozotocin-Induced Diabetic Rats, Journal of Diabetes Research. (2015) 2015, no. 1, 390428, 10.1155/2015/390428, 2-s2.0-84940392509, 26347890. 26347890 PMC4549548

